# Gene model for the ortholog of *gig* in *Drosophila ananassae*

**DOI:** 10.17912/micropub.biology.001031

**Published:** 2026-07-06

**Authors:** Anne E. Backlund, Logan Cohen, Gregory Sileo, Ian Alberts, Thomas C. Giarla, Chinmay P. Rele, Laura K. Reed

**Affiliations:** 1 The University of Alabama, Tuscaloosa, AL USA; 2 Worcester State University, Worcester MA, USA; 3 Siena College, Loudonville, NY USA; 4 University of Evansville, Evansville IN USA

## Abstract

Gene model for the ortholog of gigas ( *gig* ) in the *D. ananassae* May 2011 (Agencourt dana_caf1/DanaCAF1) Genome Assembly (GenBank Accession: GCA_000005115.1 ). This ortholog was characterized as part of a developing dataset to study the evolution of the Insulin/insulin-like growth factor signaling pathway (IIS) across the genus *Drosophila* using the Genomics Education Partnership gene annotation protocol for Course-based Undergraduate Research Experiences.

**Figure 1. Genomic neighborhood and gene model for gig in Drosophila ananassae:** **
(A) Synteny comparison of the genomic neighborhoods for *gig* in *Drosophila melanogaster* and *D. ananassae* .** Thin underlying arrows indicate the DNA strand within which the target gene— *gig* —is located in *D. melanogaster* (top) and *D. ananassae* (bottom). The thin arrows pointing to the right indicate that *gig* is on the positive (+) strand in *D. ananassae* and *D. melanogaster* . The wide gene arrows pointing in the same direction as *gig* are on the same strand relative to the thin underlying arrows, while wide gene arrows pointing in the opposite direction of *gig* are on the opposite strand relative to the thin underlying arrows. White gene arrows in *D. ananassae* indicate orthology to the corresponding gene in *D. melanogaster* , while black gene arrows indicate non-orthology. Gene symbols given in the *D. ananassae* gene arrows indicate the orthologous gene in *D. melanogaster* , while the locus identifiers are specific to *D. ananassae* . **(B) Gene Model in GEP UCSC Track Data Hub** (Raney et al., 2014). The coding-regions of *gig* in *D. ananassae* are displayed in the User Supplied Track (black); coding CDSs are depicted by thick rectangles and introns by thin lines with arrows indicating the direction of transcription. Subsequent evidence tracks include BLAT Alignments of NCBI RefSeq Genes (dark blue, alignment of Ref-Seq genes for *D. ananassae* ), Spaln of *D. melanogaster* Proteins (purple, alignment of Ref-Seq proteins from *D. melanogaster* ), Transcripts and Coding Regions Predicted by TransDecoder (dark green), RNA-Seq from Adult Females, Adult Males, and *Wolbachia* cured Embryos (red, light blue, and pink, respectively; alignment of Illumina RNA-Seq reads from *D. ananassae* ), and Splice Junctions Predicted by regtools using *D. ananassae* RNA-Seq ( SRP006203 , SRP007906 , PRJNA257286 , PRJNA388952 ). Splice junctions shown have read-depths of 100-499 and 500-999 supporting reads in pink or brown, respectively. **(C) Dot Plot of gig-PA in *D. melanogaster* ( *x* -axis) vs. the orthologous peptide in *D. ananassae* ( *y* -axis).** Amino acid number is indicated along the left and bottom; CDS number is indicated along the top and right, and CDSs are also highlighted with alternating colors. Line breaks in the dot plot indicate mismatching amino acids at the specified location between species. (D) **Tenth CDS of** **Gene Model in GEP UCSC Track Data Hub** (Raney et al., 2014). The same evidence tracks as Figure 1B are displayed, in addition to the TBLASTN Mapping of *D. melanogaster* CDS track. Isoforms *gig-RA* and *gig-RB* differ only by the length of the tenth coding CDS: *gig-RB* is slightly shorter than *gig-RA* . Although there is only one prediction in the BLAT Alignment of NCBI RefSeq Genes track, the presence of the splice acceptor (AG) in the conserved position (red box) suggests that isoform *gig-RB* also exists in *D. ananassae* .

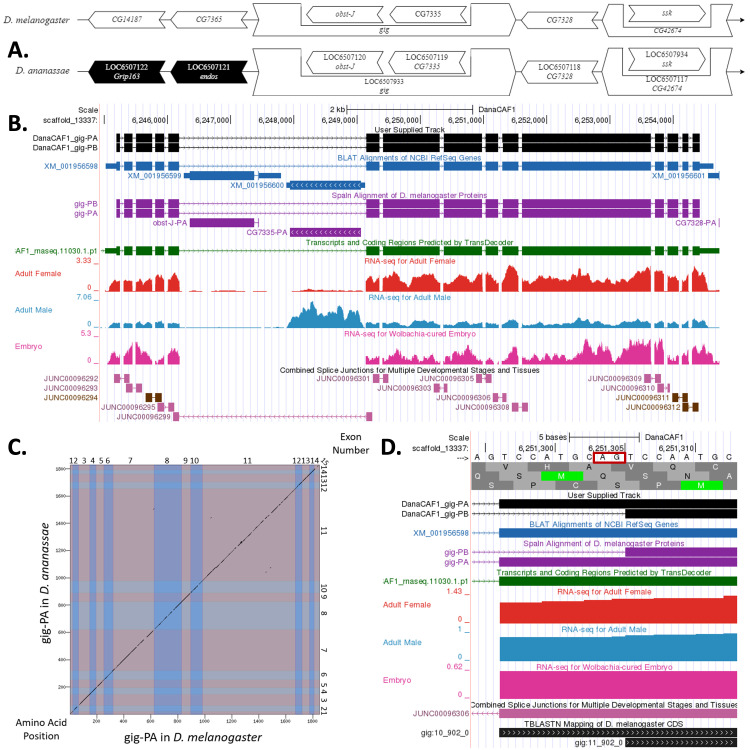



## Description

**Table:** 

* This article reports a predicted gene model generated by undergraduate work using a structured gene model annotation protocol defined by the Genomics Education Partnership (GEP; thegep.org ) for Course-based Undergraduate Research Experience (CURE). The following information in this box may be repeated in other articles submitted by participants using the same GEP CURE protocol for annotating Drosophila species orthologs of Drosophila melanogaster genes in the insulin signaling pathway. * "In this GEP CURE protocol students use web-based tools to manually annotate genes in non-model *Drosophila* species based on orthology to genes in the well-annotated model organism fruitfly *Drosophila melanogaster* . The GEP uses web-based tools to allow undergraduates to participate in course-based research by generating manual annotations of genes in non-model species (Rele et al., 2023). Computational-based gene predictions in any organism are often improved by careful manual annotation and curation, allowing for more accurate analyses of gene and genome evolution (Mudge and Harrow 2016; Tello-Ruiz et al., 2019). These models of orthologous genes across species, such as the one presented here, then provide a reliable basis for further evolutionary genomic analyses when made available to the scientific community.” (Myers et al., 2024). “The particular gene ortholog described here was characterized as part of a developing dataset to study the evolution of the Insulin/insulin-like growth factor signaling pathway (IIS) across the genus *Drosophila* . The Insulin/insulin-like growth factor signaling pathway (IIS) is a highly conserved signaling pathway in animals and is central to mediating organismal responses to nutrients (Hietakangas and Cohen 2009; Grewal 2009).” (Myers et al., 2024). “Gene *gig* (short for gigas, aka *TSC2* , *CG697* 5, FBgn0005198) encodes a tumor suppressor protein that controls cell size, cell proliferation, and organ size (Ito and Rubin 1999). The gig protein contains a GTPase-activating protein (GAP) domain and forms a complex with protein Tsc1 (Gao et al., 2001; Potter et al., 2001). The gig-Tsc1 protein complex promotes GTP hydrolysis of the small G-protein Rheb (Ras homolog enriched in brain), thereby antagonizing the insulin and TOR signaling pathways (Gao et al., 2002; Zhang et al., 2003). This gene was originally identified in humans as TSC2, and mutations in TSC1 or TSC2 result in tuberous sclerosis, a syndrome characterized by widespread benign tumors (European Consortium 1993; van Slegtenhorst et al., 1997).” (Gruys et al., 2025b). “ *D* . *ananassae* (NCBI:txid7217) is part of the *melanogaster* species group within the subgenus *Sophophora* of the genus *Drosophila* (Sturtevant 1939; Bock and Wheeler 1972). It was first described by Doleschall (1858). *D. ananassae* is circumtropical (Markow and O'Grady 2005; https://www.taxodros.uzh.ch, accessed 1 Feb 2023), and often associated with human settlement (Singh 2010). It has been extensively studied as a model for its cytogenetic and genetic characteristics, and in experimental evolution (Kikkawa 1938; Singh and Yadav 2015).” (Lawson et al., 2024).

We propose a gene model for the *D. ananassae* ortholog of the *D. melanogaster* gigas ( *gig* ) gene. The genomic region of the ortholog corresponds to the uncharacterized protein XP_001956634.1 (Locus ID LOC6507933 ) in the May 2011 (Agencourt dana_caf1/DanaCAF1) Genome Assembly of *D. ananassae* ( GCA_000005115.1 ; Drosophila 12 Genomes Consortium et al., 2007). This model is based on RNA-Seq data from *D. ananassae* ( SRP006203 , SRP007906 , PRJNA257286 , PRJNA388952 ; Graveley et al., 2011) and *gig* in *D. melanogaster* using FlyBase release FB2023_03 ( GCA_000001215.4 ; Larkin et al., 2021; Gramates et al., 2022).

***Synteny***

The reference gene, *gig,* occurs on chromosome 3L in *D. melanogaster* and is flanked upstream by *CG14187* and *CG7365* and downstream by *CG7328* and *CG42674* . The genes *obstructor-J* ( *obst-J* ) and *CG7335* are nested within *gig* , and *Snakeskin* ( *Ssk* ) is nested within *CG42674* . The *tblastn* search of *D. melanogaster* gig-PA (query) against the *D. ananassae* (GCA000005115.1) Genome Assembly (database) placed the putative ortholog of *gig* within scaffold_13337 at locus LOC6507933 ( XP_001956634.1 )— with an E-value of 0.0 and a percent identity of 68.20%. Furthermore, the putative ortholog is flanked upstream by LOC6507122 ( XP_001956632.1 ) and LOC6507121 ( XP_044570979.1 ), which correspond to *Grip163* and *endos ulfine* ( *endos* ) in *D. melanogaster* (E-value: 0.0 and 3e-83; identity: 54.05% and 97.48%, respectively, as determined by *blastp* ; Figure 1A ; Altschul et al., 1990). The putative ortholog of *gig* is flanked downstream by LOC6507118 ( XP_001956637.1 ) and LOC6507117 ( XP_032310418.1 ), which correspond to *CG7328* and *CG42674* in *D. melanogaster* (E-value: 1e-161 and 0.0; identity: 68.54% and 82.06%, respectively, as determined by *blastp* ). LOC6507120 ( XP_001956635.1 ) and LOC6507119 ( XP_001956636.1 ) are nested within *gig* (E-value: 1e-143 and 2e-170; identity: 53.95% and 65.33%). LOC6507934 ( XP_001956640.1 ) is nested within *CG42674* (E-value: 8e-115, identity: 98.77%). The putative ortholog assignment for *gig* in *D. ananassae* is supported by the following evidence: The genes downstream of and nested within the *gig* ortholog are orthologous to the genes at the same locus in *D. melanogaster* and local synteny is mostly conserved, supported by results generated from *blastp* , so we conclude that LOC6507933 is the correct ortholog of *gig* in *D. ananassae* ( Figure 1A ).

***Protein Model***

*gig* in *D. melanogaster* has two protein-coding isoforms (gig-PA and gig-PB; Figure 1B ). Both mRNA isoforms contain fifteen protein-coding CDSs, but *gig-RB* has a slightly shorter tenth CDS than *gig-RA* . The isoform and protein-coding CDS counts are conserved in *D. ananassae* . The sequence of gig-PA in *D. ananassae* has 87.67% identity (E-value: 0.0) with the protein-coding isoform gig-PA in *D. melanogaster* , as determined by *blastp* ( Figure 1C ). Coordinates of this curated gene model (gig-PB, gig-PA) are stored by NCBI at GenBank/BankIt (accession **BK064615 , BK064616** , respectively). These data are also archived in the CaltechDATA repository (see “Extended Data” section below).

***Special characteristics of the protein model***

Although there is only one prediction in the BLAT Alignment of NCBI RefSeq Genes track ( Figure 1B ), there is evidence that both isoforms of *gig* are present in *D. ananassae* . *gig-RA* and *gig-RB* differ only by the length of their tenth CDS: *gig-RB* 's CDS is slightly shorter than *gig-RA* . There is a splice acceptor site (AG) in the same reading frame that *gig-RA* uses ( Figure 1D ), which suggests that isoform *gig-RB* exists in *D. ananassae* . The TBLASTN Mapping of *D. melanogaster* CDS track also supports the presence of *gig-RB* .

## Methods

Detailed methods including algorithms, database versions, and citations for the complete annotation process can be found in Rele et al. (2023). Briefly, students use the GEP instance of the UCSC Genome Browser v.435 ( https://gander.wustl.edu ; Kent WJ et al., 2002; Navarro Gonzalez et al., 2021) to examine the genomic neighborhood of their reference IIS gene in the *D. melanogaster* genome assembly (Aug. 2014; BDGP Release 6 + ISO1 MT/dm6). Students then retrieve the protein sequence for the *D. melanogaster* reference gene for a given isoform and run it using *tblastn* against their target *Drosophila* species genome assembly on the NCBI BLAST server ( https://blast.ncbi.nlm.nih.gov/Blast.cgi ; Altschul et al., 1990) to identify potential orthologs. To validate the potential ortholog, students compare the local genomic neighborhood of their potential ortholog with the genomic neighborhood of their reference gene in *D. melanogaster* . This local synteny analysis includes at minimum the two upstream and downstream genes relative to their putative ortholog. They also explore other sets of genomic evidence using multiple alignment tracks in the Genome Browser, including BLAT alignments of RefSeq Genes, Spaln alignment of *D. melanogaster* proteins, multiple gene prediction tracks (e.g., GeMoMa, Geneid, Augustus), and modENCODE RNA-Seq from the target species. Detailed explanation of how these lines of genomic evidenced are leveraged by students in gene model development are described in Rele et al. (2023). Genomic structure information (e.g., CDSs, intron-exon number and boundaries, number of isoforms) for the *D. melanogaster* reference gene is retrieved through the Gene Record Finder ( https://gander.wustl.edu/~wilson/dmelgenerecord/index.html ; Rele et al *.,* 2023). Approximate splice sites within the target gene are determined using *tblastn* using the CDSs from the *D. melanogaste* r reference gene. Coordinates of CDSs are then refined by examining aligned modENCODE RNA-Seq data, and by applying paradigms of molecular biology such as identifying canonical splice site sequences and ensuring the maintenance of an open reading frame across hypothesized splice sites. Students then confirm the biological validity of their target gene model using the Gene Model Checker ( https://gander.wustl.edu/~wilson/genechecker/index.html ; Rele et al., 2023), which compares the structure and translated sequence from their hypothesized target gene model against the *D. melanogaster* reference gene model. At least two independent models for a gene are generated by students under mentorship of their faculty course instructors. Those models are then reconciled by a third independent researcher mentored by the project leaders to produce the final model. Note: comparison of 5' and 3' UTR sequence information is not included in this GEP CURE protocol (Gruys et al., 2025a).

## Data Availability

Description: Zip file containing FASTA, PEP, and GFF of the model. Resource Type: Model. DOI: https://doi.org/10.22002/am768-qep80

## References

[R1] Altschul SF, Gish W, Miller W, Myers EW, Lipman DJ (1990). Basic local alignment search tool.. J Mol Biol.

[R2] Bock IR, Wheeler MR. (1972). The Drosophila melanogaster species group. Univ. Texas Publs Stud. Genet. 7(7213): 1-102. FBrf0024428

[R3] Doleschall CL. 1858. Derde bijdrage tot de kennis der Dipteren fauna van nederlandsch indie. Natuurk. Tijd. Ned.-Indie 17: 73-128. FBrf0000091

[R4] Clark AG, Eisen MB, Smith DR, Bergman CM, Oliver B, Markow TA, Kaufman TC, Kellis M, Gelbart W, Iyer VN, Pollard DA, Sackton TB, Larracuente AM, Singh ND, Abad JP, Abt DN, Adryan B, Aguade M, Akashi H, Anderson WW, Aquadro CF, Ardell DH, Arguello R, Artieri CG, Barbash DA, Barker D, Barsanti P, Batterham P, Batzoglou S, Begun D, Bhutkar A, Blanco E, Bosak SA, Bradley RK, Brand AD, Brent MR, Brooks AN, Brown RH, Butlin RK, Caggese C, Calvi BR, Bernardo de Carvalho A, Caspi A, Castrezana S, Celniker SE, Chang JL, Chapple C, Chatterji S, Chinwalla A, Civetta A, Clifton SW, Comeron JM, Costello JC, Coyne JA, Daub J, David RG, Delcher AL, Delehaunty K, Do CB, Ebling H, Edwards K, Eickbush T, Evans JD, Filipski A, Findeiss S, Freyhult E, Fulton L, Fulton R, Garcia AC, Gardiner A, Garfield DA, Garvin BE, Gibson G, Gilbert D, Gnerre S, Godfrey J, Good R, Gotea V, Gravely B, Greenberg AJ, Griffiths-Jones S, Gross S, Guigo R, Gustafson EA, Haerty W, Hahn MW, Halligan DL, Halpern AL, Halter GM, Han MV, Heger A, Hillier L, Hinrichs AS, Holmes I, Hoskins RA, Hubisz MJ, Hultmark D, Huntley MA, Jaffe DB, Jagadeeshan S, Jeck WR, Johnson J, Jones CD, Jordan WC, Karpen GH, Kataoka E, Keightley PD, Kheradpour P, Kirkness EF, Koerich LB, Kristiansen K, Kudrna D, Kulathinal RJ, Kumar S, Kwok R, Lander E, Langley CH, Lapoint R, Lazzaro BP, Lee SJ, Levesque L, Li R, Lin CF, Lin MF, Lindblad-Toh K, Llopart A, Long M, Low L, Lozovsky E, Lu J, Luo M, Machado CA, Makalowski W, Marzo M, Matsuda M, Matzkin L, McAllister B, McBride CS, McKernan B, McKernan K, Mendez-Lago M, Minx P, Mollenhauer MU, Montooth K, Mount SM, Mu X, Myers E, Negre B, Newfeld S, Nielsen R, Noor MA, O'Grady P, Pachter L, Papaceit M, Parisi MJ, Parisi M, Parts L, Pedersen JS, Pesole G, Phillippy AM, Ponting CP, Pop M, Porcelli D, Powell JR, Prohaska S, Pruitt K, Puig M, Quesneville H, Ram KR, Rand D, Rasmussen MD, Reed LK, Reenan R, Reily A, Remington KA, Rieger TT, Ritchie MG, Robin C, Rogers YH, Rohde C, Rozas J, Rubenfield MJ, Ruiz A, Russo S, Salzberg SL, Sanchez-Gracia A, Saranga DJ, Sato H, Schaeffer SW, Schatz MC, Schlenke T, Schwartz R, Segarra C, Singh RS, Sirot L, Sirota M, Sisneros NB, Smith CD, Smith TF, Spieth J, Stage DE, Stark A, Stephan W, Strausberg RL, Strempel S, Sturgill D, Sutton G, Sutton GG, Tao W, Teichmann S, Tobari YN, Tomimura Y, Tsolas JM, Valente VL, Venter E, Venter JC, Vicario S, Vieira FG, Vilella AJ, Villasante A, Walenz B, Wang J, Wasserman M, Watts T, Wilson D, Wilson RK, Wing RA, Wolfner MF, Wong A, Wong GK, Wu CI, Wu G, Yamamoto D, Yang HP, Yang SP, Yorke JA, Yoshida K, Zdobnov E, Zhang P, Zhang Y, Zimin AV, Baldwin J, Abdouelleil A, Abdulkadir J, Abebe A, Abera B, Abreu J, Acer SC, Aftuck L, Alexander A, An P, Anderson E, Anderson S, Arachi H, Azer M, Bachantsang P, Barry A, Bayul T, Berlin A, Bessette D, Bloom T, Blye J, Boguslavskiy L, Bonnet C, Boukhgalter B, Bourzgui I, Brown A, Cahill P, Channer S, Cheshatsang Y, Chuda L, Citroen M, Collymore A, Cooke P, Costello M, D'Aco K, Daza R, De Haan G, DeGray S, DeMaso C, Dhargay N, Dooley K, Dooley E, Doricent M, Dorje P, Dorjee K, Dupes A, Elong R, Falk J, Farina A, Faro S, Ferguson D, Fisher S, Foley CD, Franke A, Friedrich D, Gadbois L, Gearin G, Gearin CR, Giannoukos G, Goode T, Graham J, Grandbois E, Grewal S, Gyaltsen K, Hafez N, Hagos B, Hall J, Henson C, Hollinger A, Honan T, Huard MD, Hughes L, Hurhula B, Husby ME, Kamat A, Kanga B, Kashin S, Khazanovich D, Kisner P, Lance K, Lara M, Lee W, Lennon N, Letendre F, LeVine R, Lipovsky A, Liu X, Liu J, Liu S, Lokyitsang T, Lokyitsang Y, Lubonja R, Lui A, MacDonald P, Magnisalis V, Maru K, Matthews C, McCusker W, McDonough S, Mehta T, Meldrim J, Meneus L, Mihai O, Mihalev A, Mihova T, Mittelman R, Mlenga V, Montmayeur A, Mulrain L, Navidi A, Naylor J, Negash T, Nguyen T, Nguyen N, Nicol R, Norbu C, Norbu N, Novod N, O'Neill B, Osman S, Markiewicz E, Oyono OL, Patti C, Phunkhang P, Pierre F, Priest M, Raghuraman S, Rege F, Reyes R, Rise C, Rogov P, Ross K, Ryan E, Settipalli S, Shea T, Sherpa N, Shi L, Shih D, Sparrow T, Spaulding J, Stalker J, Stange-Thomann N, Stavropoulos S, Stone C, Strader C, Tesfaye S, Thomson T, Thoulutsang Y, Thoulutsang D, Topham K, Topping I, Tsamla T, Vassiliev H, Vo A, Wangchuk T, Wangdi T, Weiand M, Wilkinson J, Wilson A, Yadav S, Young G, Yu Q, Zembek L, Zhong D, Zimmer A, Zwirko Z, Jaffe DB, Alvarez P, Brockman W, Butler J, Chin C, Gnerre S, Grabherr M, Kleber M, Mauceli E, MacCallum I, Drosophila 12 Genomes Consortium. (2007). Evolution of genes and genomes on the Drosophila phylogeny.. Nature.

[R5] European Chromosome 16 Tuberous Sclerosis Consortium (1993). Identification and characterization of the tuberous sclerosis gene on chromosome 16.. Cell.

[R6] Gao X, Pan D (2001). TSC1 and TSC2 tumor suppressors antagonize insulin signaling in cell growth.. Genes Dev.

[R7] Gao X, Zhang Y, Arrazola P, Hino O, Kobayashi T, Yeung RS, Ru B, Pan D (2002). Tsc tumour suppressor proteins antagonize amino-acid-TOR signalling.. Nat Cell Biol.

[R8] Gramates L Sian, Agapite Julie, Attrill Helen, Calvi Brian R, Crosby Madeline A, dos Santos Gilberto, Goodman Joshua L, Goutte-Gattat Damien, Jenkins Victoria K, Kaufman Thomas, Larkin Aoife, Matthews Beverley B, Millburn Gillian, Strelets Victor B, Perrimon Norbert, Gelbart Susan Russo, Agapite Julie, Broll Kris, Crosby Lynn, dos Santos Gil, Falls Kathleen, Gramates L Sian, Jenkins Victoria, Longden Ian, Matthews Beverley, Seme Jolene, Tabone Christopher J, Zhou Pinglei, Zytkovicz Mark, Brown Nick, Antonazzo Giulia, Attrill Helen, Garapati Phani, Goutte-Gattat Damien, Larkin Aoife, Marygold Steven, McLachlan Alex, Millburn Gillian, Öztürk-Çolak Arzu, Pilgrim Clare, Trovisco Vitor, Calvi Brian, Kaufman Thomas, Goodman Josh, Krishna Pravija, Strelets Victor, Thurmond Jim, Cripps Richard, Lovato TyAnna, the FlyBase Consortium (2022). FlyBase: a guided tour of highlighted features. Genetics.

[R9] Graveley BR, Brooks AN, Carlson JW, Duff MO, Landolin JM, Yang L, Artieri CG, van Baren MJ, Boley N, Booth BW, Brown JB, Cherbas L, Davis CA, Dobin A, Li R, Lin W, Malone JH, Mattiuzzo NR, Miller D, Sturgill D, Tuch BB, Zaleski C, Zhang D, Blanchette M, Dudoit S, Eads B, Green RE, Hammonds A, Jiang L, Kapranov P, Langton L, Perrimon N, Sandler JE, Wan KH, Willingham A, Zhang Y, Zou Y, Andrews J, Bickel PJ, Brenner SE, Brent MR, Cherbas P, Gingeras TR, Hoskins RA, Kaufman TC, Oliver B, Celniker SE (2010). The developmental transcriptome of Drosophila melanogaster.. Nature.

[R10] Grewal SS (2008). Insulin/TOR signaling in growth and homeostasis: a view from the fly world.. Int J Biochem Cell Biol.

[R11] Gruys ML, Sharp MA, Lill Z, Xiong C, Hark AT, Youngblom JJ, Rele CP, Reed LK (2025). Gene model for the ortholog of Glys in Drosophila simulans.. MicroPubl Biol.

[R12] Gruys ML, Sharp MA, Croslyn C, Yap-Chiongco M, Reed LK, Stamm J, Rele CP (2025). Gene model for the ortholog of gig in Drosophila mojavensis.. MicroPubl Biol.

[R13] Hietakangas V, Cohen SM (2009). Regulation of tissue growth through nutrient sensing.. Annu Rev Genet.

[R14] Ito N, Rubin GM (1999). gigas, a Drosophila homolog of tuberous sclerosis gene product-2, regulates the cell cycle.. Cell.

[R15] Jenkins VK, Larkin A, Thurmond J, FlyBase Consortium (2022). Using FlyBase: A Database of Drosophila Genes and Genetics.. Methods Mol Biol.

[R16] Kent WJ, Sugnet CW, Furey TS, Roskin KM, Pringle TH, Zahler AM, Haussler D (2002). The human genome browser at UCSC.. Genome Res.

[R17] Kikkawa Hideo (1938). Studies on the genetics and cytology ofDrosophila ananassae. Genetica.

[R18] Larkin A, Marygold SJ, Antonazzo G, Attrill H, Dos Santos G, Garapati PV, Goodman JL, Gramates LS, Millburn G, Strelets VB, Tabone CJ, Thurmond J, FlyBase Consortium. (2021). FlyBase: updates to the Drosophila melanogaster knowledge base.. Nucleic Acids Res.

[R19] Lawson ME, McAbee M, Lucas RA, Tanner S, Wittke-Thompson J, Pelletier TA, Ozsoy Z, Sterne-Marr R, Rele CP, Reed LK (2024). Gene model for the ortholog of Ilp5 in Drosophila ananassae.. MicroPubl Biol.

[R20] Markow TA and O’Grady P. (2005) Drosophila: A guide to species identification and use. 978-0-12-473052-6

[R21] Mudge JM, Harrow J (2016). The state of play in higher eukaryote gene annotation.. Nat Rev Genet.

[R22] Myers A, Hoffman A, Natysin M, Arsham AM, Stamm J, Thompson JS, Rele CP, Reed LK (2024). Gene model for the ortholog Myc in Drosophila ananassae.. MicroPubl Biol.

[R23] Navarro Gonzalez J, Zweig AS, Speir ML, Schmelter D, Rosenbloom KR, Raney BJ, Powell CC, Nassar LR, Maulding ND, Lee CM, Lee BT, Hinrichs AS, Fyfe AC, Fernandes JD, Diekhans M, Clawson H, Casper J, Benet-Pagès A, Barber GP, Haussler D, Kuhn RM, Haeussler M, Kent WJ (2021). The UCSC Genome Browser database: 2021 update.. Nucleic Acids Res.

[R24] Potter CJ, Huang H, Xu T (2001). Drosophila Tsc1 functions with Tsc2 to antagonize insulin signaling in regulating cell growth, cell proliferation, and organ size.. Cell.

[R25] Raney BJ, Dreszer TR, Barber GP, Clawson H, Fujita PA, Wang T, Nguyen N, Paten B, Zweig AS, Karolchik D, Kent WJ (2013). Track data hubs enable visualization of user-defined genome-wide annotations on the UCSC Genome Browser.. Bioinformatics.

[R26] Rele Chinmay P., Sandlin Katie M., Leung Wilson, Reed Laura K. (2023). Manual annotation of Drosophila genes: a Genomics Education Partnership protocol. F1000Research.

[R27] Singh BN (2010). Drosophila ananassae: a good model species for genetical, behavioural and evolutionary studies.. Indian J Exp Biol.

[R28] Singh BN, Yadav JP (2015). Status of research on Drosophila ananassae at global level.. J Genet.

[R29] Sturtevant AH (1939). On the Subdivision of the Genus Drosophila.. Proc Natl Acad Sci U S A.

[R30] Tello-Ruiz MK, Marco CF, Hsu FM, Khangura RS, Qiao P, Sapkota S, Stitzer MC, Wasikowski R, Wu H, Zhan J, Chougule K, Barone LC, Ghiban C, Muna D, Olson AC, Wang L, Ware D, Micklos DA (2019). Double triage to identify poorly annotated genes in maize: The missing link in community curation.. PLoS One.

[R31] van Slegtenhorst M, de Hoogt R, Hermans C, Nellist M, Janssen B, Verhoef S, Lindhout D, van den Ouweland A, Halley D, Young J, Burley M, Jeremiah S, Woodward K, Nahmias J, Fox M, Ekong R, Osborne J, Wolfe J, Povey S, Snell RG, Cheadle JP, Jones AC, Tachataki M, Ravine D, Sampson JR, Reeve MP, Richardson P, Wilmer F, Munro C, Hawkins TL, Sepp T, Ali JB, Ward S, Green AJ, Yates JR, Kwiatkowska J, Henske EP, Short MP, Haines JH, Jozwiak S, Kwiatkowski DJ (1997). Identification of the tuberous sclerosis gene TSC1 on chromosome 9q34.. Science.

[R32] Zhang Y, Gao X, Saucedo LJ, Ru B, Edgar BA, Pan D (2003). Rheb is a direct target of the tuberous sclerosis tumour suppressor proteins.. Nat Cell Biol.

